# Effect of Deposition Temperature on Long-Term Residual Stress Evolution of Au Films

**DOI:** 10.3390/ma16103645

**Published:** 2023-05-10

**Authors:** Shujun Zhou, Wei Wu, Yilun Yang, Xiao Huang

**Affiliations:** 1 School of Mechanical Electronic and Information Engineering, China University of Mining and Technology (Beijing), Beijing 100083, China; 2 State Key Laboratory of Tribology, Tsinghua University, Beijing 100084, China

**Keywords:** Au film, residual stress, deposition temperature, microstructure, stability

## Abstract

To increase the residual stress stability of Au films while reducing the residual stress level, the effect of deposition temperature on long-term residual stress evolution of Au films under different conditions were studied. Au films with a thickness of 360 nm were deposited using e-beam evaporation on fused silica under different temperatures. Observations and comparisons were made of the microstructures of Au films deposited under different temperatures. Results showed that by increasing the deposition temperature, a more compact microstructure of Au film was obtained, which was manifested in increased grain size and reduced grain-boundary voids. After deposition, a combined process consisting of natural placement and 80 °C thermal holding was conducted on the Au films, and the residual stresses were monitored using the curvature-based technique. Results showed that the initial tensile residual stress of the as-deposited film decreased with the deposition temperature. The Au films with higher deposition temperatures showed better residual stress stability, maintaining low stress levels in the subsequent long-term combination of natural placement and thermal holding. The mechanism was discussed based on the differences in microstructure. Comparisons were made between post-deposition annealing and increased deposition temperature.

## 1. Introduction

Residual stress inevitably exists in thin films and is a key source of instability in their use [1,2,3,4,5]. The residual stress can cause rupturing, wrinkles, or even delamination of the thin films. When the substrate thickness is relatively low, the presence and variation of residual stress will cause structure deflection or distortion, resulting in structural instability. Generally, the residual stress of thin films is composed of thermal stress and intrinsic stress [1,2]. The coefficients of thermal expansion (CTE) of film and substrate materials are usually different. As the temperature varies, thermal stress is generated due to the thermal mismatch. The origin of intrinsic stress depends strongly on the film and substrate materials, as well as the processing parameters of the film deposition.

Au films are extensively used in various electronic and optical devices [6,7]. However, the residual stress in Au films is subject to change. For example, tensile stress may increase under thermal holding (TH) and decrease under natural placement (NP) [8]. The structural instability caused by residual stress variations can lead to degraded performance of the device. Hence, exploring the behavior and control of residual stress in Au films is crucial for their widespread use.

For better application of Au films, tremendous efforts were made to study and control residual stress. Some researchers focused on the deposition process, including the effects of the deposition method, deposition rate, temperature, pressure, and current density on the origins of residual stress in Au film during deposition. Thornell et al. [9] studied residual stress in magnetron-sputtered Au film under varying parameters. Results showed that the residual stress of the as-deposited film was compressive, and a lower deposition pressure or higher deposition rate resulted in a higher value of compressive stress. Kebabi [10] and Malek [11] explored the residual stress in Au film deposited by different methods and under various parameters. According to their results, electroplated Au films had the lowest stress values, the electron beam-evaporated Au films had intermediate stress values, and the diode-sputtered Au films showed the highest stress values. Lower current density and lower temperature corresponded to reduced residual stress. Qiu et al. [12] studied Au films sputtered on SiO_2_/Si(100) and mica. Results showed that the films deposited on SiO_2_/Si(100) displayed tensile stress, while on mica the stress was compressive for **[111]** and **[311]** orientation grains and tensile stress for **[200]** and **[220]** orientation grains. The mechanisms of the stress origin and its variation were closely related to the microstructure evolution of the films during deposition. The Au films fabricated by various methods generally showed a polycrystalline structure. Over recent decades, comprehensive understanding has been reached on the mechanism of the origin of intrinsic stress in thin polycrystalline films [13,14,15,16,17,18]. Regarding the different stages of the film growth, the proposed mechanisms mainly include compressive stress before island (grain) coalescence [13,14], tensile stress during island (grain) contiguity [13,14,15,16], and compressive stress after island coalescence during the continued growth of the film [13,17,18]. Abadias [16] sputter-deposited Au film on Si wafers covered with native oxide (SiOx), observed the complex compressive –tensile–compressive stress behavior, and provided direct evidence that the film continuity coincides with the tensile stress maximum during the deposition process.

Some studies focused on the influence of post-deposition thermal treatment on the residual stress of gold films. The proposed mechanisms for residual stress variations under post-deposition heat treatment include texture evolution, grain growth, defect annihilation, grain grooving, and solid solution behavior. Tang et al. [19] studied the annealing of Au films deposited on Al_2_O_3_ substrate at 400 °C, indicating that the residual stress changed from tension to compression, and that the residual stress variation can be associated with the decrease in the (111)/(100) ratio of diffraction peak intensity (texture evolution). Zhang et al. [20] explained the phenomenon of the abnormal growth of (100) oriented grains and the texture evolution from (111) to (100) after post-deposition annealing from the perspective of strain energy minimization. Some researchers maintain that the increase in tensile residual stress in the post-deposition thermal treatment of Au films can be attributed to grain growth [21,22,23,24]. Due to the lower density of the grain boundary than the interior of the grain, the grain growth (partial removal of the grain boundary) will cause volume contraction in the film. Since the film is confined on the substrate, tensile stress is generated [25]. The deposition of film is usually a nonequilibrium process, in which numerous defects are formed. Defect annihilation causes volumetric subtraction in the film during post-deposition thermal treatment and was also believed to cause increased tensile stress in Au films [8,22,23,26]. Grain grooving is a typical volumetric addition process which causes expansion of the film plane, resulting in the increase in compressive stress (or decreases the tensile stress). The tensile stress reduction in Au films under thermal treatment for a relatively long time or at a high temperature [22,24,26,27] was thought to be caused by grain grooving. Zhang et al. [27] studied annealed Au films at 300 °C for 5 h, and found that the Cr diffused from the transition layer to the Au surface and formed a solid solution, resulting in increased tensile stress. During the formation of the solid solution, the Au atoms were replaced by Cr atoms, leading to volumetric subtraction. Katz et al. [21] studied the residual stress in Au films on soda–lime glass during the thermal cycling. The results showed that the tensile residual stress tended to decrease at temperatures higher than 350 °C due to the Au–Si eutectic reaction.

Though there have been valuable insights into the residual stress in Au films, the majority of the existing studies focus on the short-term behavior of residual stress during heat treatment. Few studies were conducted on the subsequent long-term behavior of the residual stress under conditions similar to actual conditions (TH alternating with NP). In our previous study [28], we found that post-deposition annealing increased the subsequent long-term stability of residual stress in Au films. However, the stress level also increased. Additionally, this may bring new reliability concerns, such as rupturing or debonding of the film. Therefore, in this paper, we aim to explore whether we can improve long-term stability while reducing the level of stress.

In this paper, we investigate the effect of deposition temperature on the subsequent long-term residual stress evolution in Au films. The effectiveness of temperature control during deposition on long-term residual stress stability in Au films is verified, and the mechanism is discussed based on the microstructure analysis.

## 2. Materials and Methods

Au films were deposited using e-beam evaporation on fused silica (30 mm × 5 mm × 0.2 mm). The silica substrate was first cleaned ultrasonically with acetone, ethanol, boiling nitric acid, diluted sodium hydroxide, and deionized water, followed by plasma for 10 min. The samples were mounted on a rotating holder to improve the consistency. The distance from samples to the crucible was about 30 cm. The base pressure for the deposition was 7 × 10^−4^ Pa. The deposition rate was maintained within the range 2.5–3 Å/s. The thickness of the Au film was monitored using a crystal quartz gauge, and verified from the cross-sectional micrographs. The thickness of the Au film was approximately 360 nm, with 10 nm of chromium serving as a bonding layer between the Au and the fused silica.

**Figure 1:**
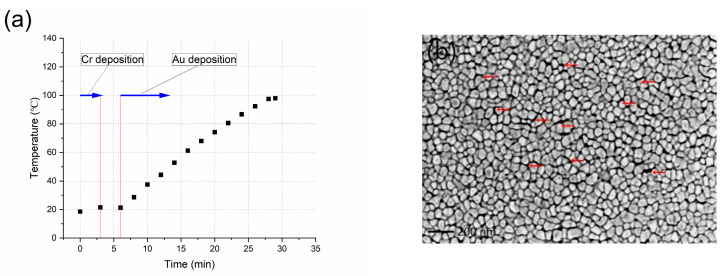

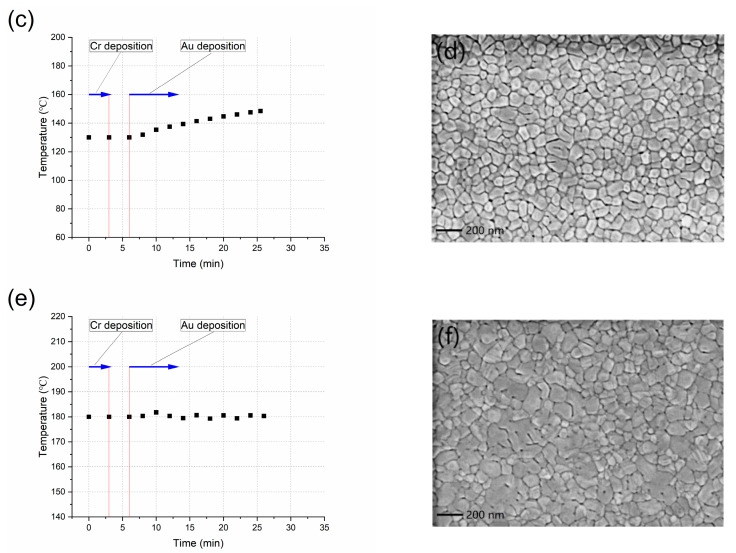
Deposition temperatures and corresponding SEM micrographs. (**a**,**b**) No preheating. (**c**,**d**) 130 °C preheating. (**e**,**f**) 180 °C preheating. The black squares and red lines in (**a**,**c**,**e**) indicate the deposition process of Cr and Au and the period of transition. The arrows in (**b**) indicates the voids distributed at grain boundaries.

Three different temperature conditions, namely, no preheating (T_d1_), 130 °C preheating (T_d2_), and 180 °C preheating (T_d3_) were utilized on the substrate. Critical diffusion of chromium from the bonding layer was observed under annealing at 225 °C [22]. To avoid this, higher temperatures were not adopted in the present study. A thermocouple was used to record the temperature of the substrate. Figure 1a,c,e illustrates the temperature variations during the deposition process. Under the conditions of no preheating (Figure 1a) and 130 °C preheating (Figure 1c), the temperature increased and varied within a certain range due to the heat of the deposition process. When the preheating temperature reached 180 °C (Figure 1e), the deposition temperature was maintained at 180 °C throughout the entire process.

The residual stress was evaluated using the curvature-based technique. Briefly, the substrate curvature radius (R_0_) before deposition and the film/substrate radius (R_1_) after deposition or processing were measured, and then the residual stress in the film was obtained through the variation of curvature and mechanics principle employing the Stoney Equation [29]:

(1) $$\sigma =\frac{E_{s}}{6\left(1-v_{s}\right)}\frac{t_{s}^{2}}{t_{f}}\left(\frac{1}{R_{1}}-\frac{1}{R_{0}}\right)$$

where $t_{s}$ is the thickness of the substrate, $t_{f}$ is the thickness of the film, $E_{s}$ is the Young’s modulus of the substrate, and $v_{s}$ is the Poisson’s ratio of the substrate.

The radius of curvature to calculate the residual stress was measured using an interferometric microscope (Zygo—Nexview). The microstructure of Au film was observed from the surface using an SEM (scanning electron microscope, Zeiss—Supra 55) operated at a low voltage of 0.5 kV. A cross-sectional sample of the Au film with no preheating was prepared using an FIB (focused ion beam, Tescan—Lyra 3) and observed using a TEM (transmission electron microscope, JEOL—JEM2011).

A process consisting of NP and TH (80 °C) was implemented to simulate long-term and complex conditions similar to the actual operation process. The detailed schedule is presented in Table 1. The residual stress of the as-deposited film was evaluated immediately after deposition (on the same day as deposition). The residual stress during the process was monitored. All residual stress measurements were performed at room temperature.

## 3. Results

### 3.1. Microstructure of Au Film Deposited under Different Temperature Conditions

**Figure 2:**
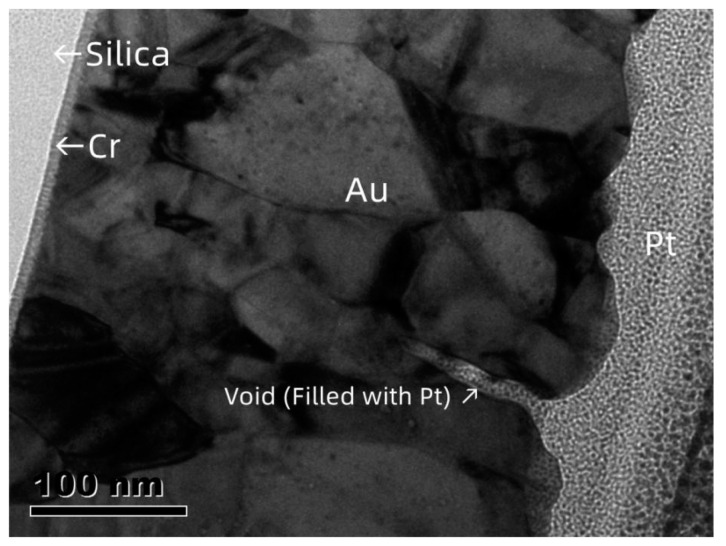
Cross-sectional TEM micrograph of Au film deposited with no preheating.

Figure 1b,d,f illustrates that the typical SEM micrographs correspond to the deposition temperature (Figure 1a,c,e). The Au film without preheating is shown in Figure 1b. The Au film shows a polycrystalline microstructure, with a grain size of approximately 50–100 nm; a large number of voids (indicated by the red arrows) are located at the grain boundary. Figure 2 illustrates the cross-sectional TEM micrograph of Au film deposited with no preheating (from another batch with identical deposition conditions). From the cross-sectional micrographs, a grain boundary void can be clearly seen. A Pt capping layer was deposited before the preparation of the TEM sample to protect the Au surface. The entry of Pt from the capping layer into the void was confirmed by energy-dispersive X-ray spectrometry, verifying the existence of the void. The microstructure of Au film without preheating is consistent with that of polycrystalline film deposited at a relatively low homologous temperature [30] (T/T_m_ < 0.3, T_m_ is the melting point of the film material, 1337 K for Au).

The microstructure of the Au film when the preheating temperature is set at 130 °C is shown in Figure 1d. Compared with the condition without preheating, the grain size increased, the voids at the grain boundaries greatly declined, and the film showed a more compact structure. This indicates that increasing the deposition temperature promotes the growth and coalescence of the grains. The microstructure of the Au film at a preheating temperature of 180 °C is shown in Figure 1f. A larger grain size can be observed, and a further compacted microstructure is shown.

### 3.2. Residual Stress Evolution

**Figure 3:**
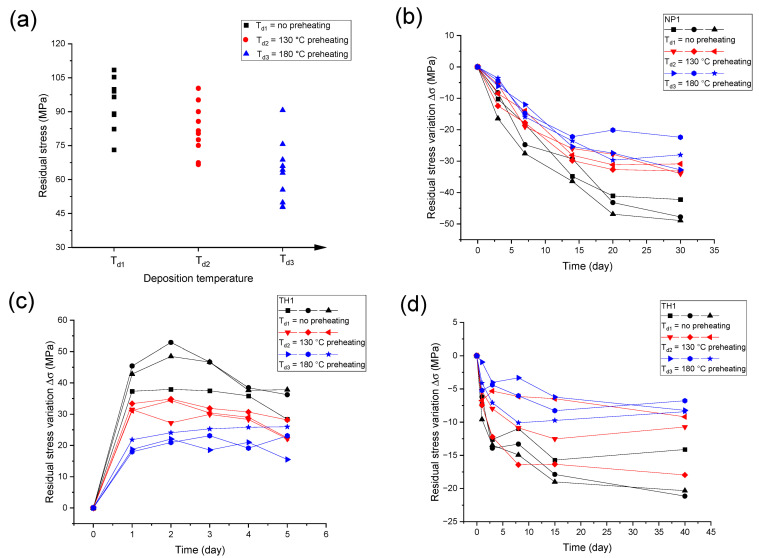
Residual stress of Au film and variation during different processes. (**a**) Stress in the as-deposited state. (**b**–**d**) Stress variations during NP1, TH1, and NP2.

Figure 3 presents the initial stress of Au films in the as-deposited state and the stress variation during the subsequent processes. For each deposition temperature condition, ten samples of Au films were deposited. Figure 3a presents the residual stress of the as-deposited Au films deposited under different temperature conditions. It can be seen that the residual stress in the as-deposited Au films dispersed in a certain range. Residual stress in the as-deposited film diminished when the deposition temperature rose. The average residual stress values are 94 ± 11 MPa in the case of no preheating, 82 ± 11 MPa in the case of 130 °C preheating, and 63 ± 14 MPa in the case of 180 °C preheating. Compared with the condition with no preheating, the average residual stress under 130 and 180 °C preheating was reduced by 13% and 33%, respectively. The sign and magnitude of residual stress in the as-deposited state for Au films were consistent with the results of Katz [21], who obtained 82 MPa in tensile stress for as-deposited Au film on cover-glass substrate.

After deposition, the complex processes consisting of NP1, TH1, and NP2 were conducted. For each deposition temperature condition, three duplicate samples were used. For a better comparison, the variations of residual stress relative to the starting point in each period are presented in Figure 3b–d, and the average value of residual stress variation with sample standard deviation is presented in Table 2. In the first natural placement (NP1), the residual stress variation is presented in Figure 3b. For all the temperature conditions, the tensile residual stress declined rapidly at the initial stage and then gradually decreased. The average values of stress decrease during NP1 are −46 ± 4 MPa in the case of no preheating, −33 ± 2 MPa in the case of 130 °C preheating, and −28 ± 5 MPa in the case of 180 °C preheating. The Au film deposited without preheating showed the largest stress decrease, while the Au film deposited at 180 °C preheating showed the smallest stress decrease. The residual stress stability during NP1 can be improved by increasing deposition temperature.

After NP1, a 5 × 1 day 80 °C thermal holding process (TH1) was performed on the Au films. The residual stress variation during TH1 is presented in Figure 3c. The residual stresses first increased, and then remained stable or decreased slightly. The maximum residual stress variations in TH1 are 46 ± 8 MPa for the case of no preheating, 34 ± 2 MPa for the case of 130 °C preheating, and 24 ± 2 MPa for the case of 180 °C preheating. With the increase in the deposition temperature, the stress variation (increase in tensile stress) decreased during TH1, and the stress stability rose.

After TH1, another natural placement process (NP2) was performed on the Au films. The residual stress variation during NP2 is presented in Figure 3d. The residual stress decreased under natural placement. The average values of stress drop in NP2 are about −19 ± 4 MPa in the case of no preheating, −13 ± 5 MPa in the case of 130 °C preheating and −8 ± 1 MPa in the case of 180 °C preheating. Likewise, the Au films deposited without preheating showed the largest stress decrease and those deposited under 180 °C preheating had the smallest stress decrease, meaning that increasing the deposition temperature can increase the residual stress stability during NP2.

**Figure 4:**
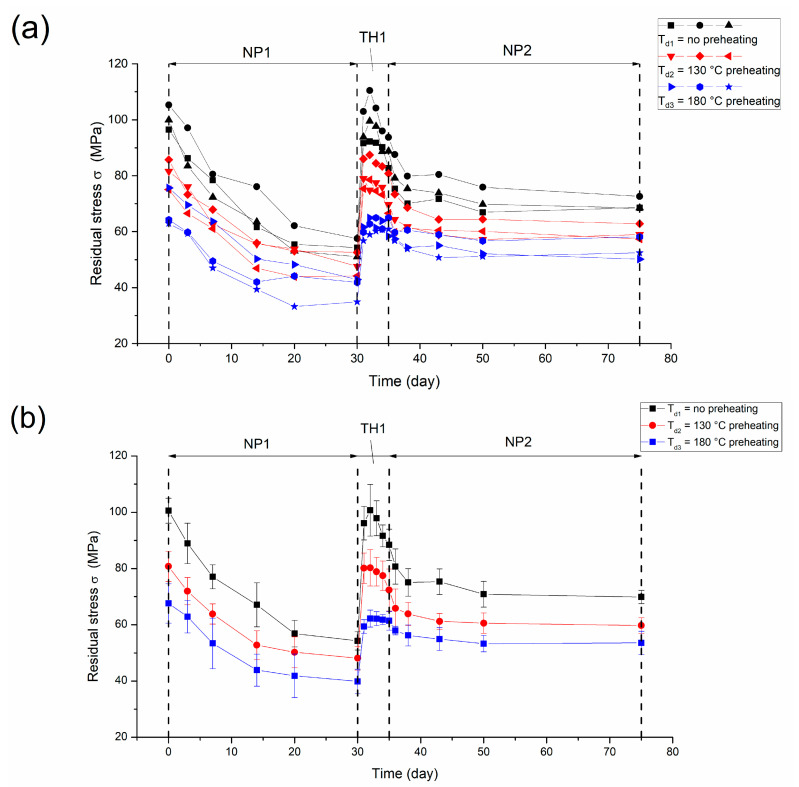
Residual stress evolution for Au films under long-term and varying conditions. (**a**) Stress in the different stages. (**b**) Average stress, with error bars.

The entire process of residual stress variations under long-term varying conditions is seen in Figure 4. In Figure 4a, it can be seen that the residual stress of Au film deposited with no preheating has the largest residual stress variation, while Au film with 180 °C preheating has minimal changes. The maximum residual stress variation (the maximum minus the minimum) for the entire process is 48 ± 5 MPa in the case of no preheating, 35 ± 0.5 MPa in the case of 130 °C preheating, and 29 ± 5 MPa in the case of 180 °C preheating. Compared to the case without preheating, the residual stress stability increased by 27% in the case of 130 °C preheating, and by 40% in the case of 180 °C preheating, on average. Figure 4b demonstrates the averages of the residual stress of Au films with error bars (sample standard deviation) in different stages.

Through the experiments above, it can be concluded that increasing deposition temperatures can both lower the stress level and boost the long-term stability of residual stress in Au film. Higher deposition temperatures show a better effect.

## 4. Discussion

### 4.1. Microstructure of Au Film Deposited under Different Temperatures

For the growth of polycrystalline film, the temperature has a substantial impact on a series of thermal activation processes and is a key factor affecting the film microstructure. According to the SEM micrographs in Figure 1, the grain size of the Au film increases with the rise of the deposition temperature. There are many reasons for the grain growth. Firstly, an increase in temperature can reduce nucleation density at the initial stage of the film formation [31]. As a result, the grain size becomes larger when the islands (grains) impinge upon each other. Secondly, the diffusivities (in bulk, at the grain boundaries and at the surface) increase with the temperature (Arrhenius dependence) [31], which can enhance the mobility of the atoms of the film materials and promote the grain coarsening process. Therefore, as the deposition temperature increases, the grain size increases.

In addition to grain sizes, a more compact microstructure with fewer voids distributed at the grain boundaries was also obtained by increasing deposition temperature. The effect of deposition temperature on the microstructure has been extensively studied and described by structure zone models [30]. Structure zones can be classified according to the homologous temperatures: zone 1 (T/T_m_ < 0.3), zone 2 (0.3 < T/T_m_ < 0.5), and zone 3 (0.5 < T/T_m_). For T/T_m_ < 0.3 (zone 1), the microstructure routinely appeared as irregular column grains and was partially separated by the voids. The Au films deposited in the present study without preheating (T/T_m_ = 0.22~0.28) mostly conform to the microstructure of zone 1. Due to the relatively low temperature and the diffusivities, the adatoms are difficult to diffuse to the “shadowed area”, and thus the voids formed at the grain boundaries are referred to as the “shadow effect”. For 0.3 < T/T_m_ < 0.5 (zone 2), due to the enhancement of the surface diffusion process, the film becomes smoother and more compact, with well-defined grain boundaries and columnar structure. The Au films deposited with 130 °C preheating (T/T_m_ = 0.30~0.32) and 180 °C preheating (T/Tm = 0.34) conform well to the microstructure of zone 2. With the increase in deposition temperature, the enhanced mobility of the adatoms makes it easier to migrate to favorable positions, which can greatly reduce the grain boundary voids and other defects in the film, resulting in a more compact structure. Differences of the initial microstructure of the Au films deposited at different temperatures have an important effect on the subsequent stress evolution.

### 4.2. Residual Stress Evolution of Au Film Deposited under Different Temperatures

**Figure 5:**
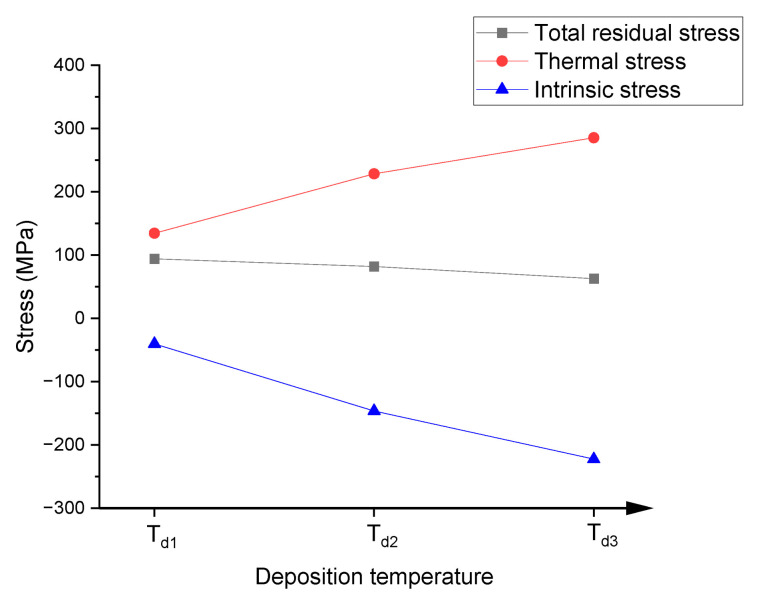
Total residual stress, thermal stress, and intrinsic stress in Au film.

According to Figure 2a, residual stress of as-deposited film decreased with the increase in deposition temperature. As already stated in the introduction, residual stress is composed of thermal stress and intrinsic stress. Thermal stress in the present study can be evaluated using the final temperature of deposition and the following equation [1]:

(2) $$\sigma =\frac{E_{f}}{1-\vartheta_{f}}\left(\alpha_{s}-\alpha_{f}\right)\left(T_{r}-T_{d}\right)$$

where, $T_{r}$ is room temperature; $T_{d}$ is the final temperature of deposition; $\alpha_{f}$ and $\alpha_{s}$ are the CTE of the Au film and the substrate, respectively; and $E_{f}$ and $\vartheta_{f}$ are Young’s modulus and Poisson’s ratio of the Au film, respectively. The parameter used in the derivation of thermal stress is listed in Table 3. The thermal stresses are 134, 228, and 286 MPa in the case of no heating, 130 °C preheating, and 180 °C preheating, respectively. Intrinsic stress can be obtained by subtracting thermal stress from total residual stress. The intrinsic stresses are −40, −146, and −223 MPa in the case of no heating, 130 °C preheating, and 180 °C preheating, respectively. The sign and magnitude of intrinsic stress in Au film are consistent with the results of Leib [32], which ranged from −70 MPa to −225 MPa for Au film of 40–200 nm. Figure 5 illustrates the total residual stress, thermal stress, and intrinsic stress of the film. The intrinsic stress decreased (more compressive) with the increase in deposition temperatures.

The effect of the deposition temperature on engendering residual stress of polycrystalline films was studied [1,2]. Firstly, raising the deposition temperature reduced the tensile stress generation during grain coalescence. During the growth of polycrystalline films, tensile stress was mainly generated at the stage of island (grain) coalescence [14]. The tensile stress was caused by the closure of the small gaps between adjacent grains prior to grain coalescence. The process was driven by the energy release of the surface area reduction. Raising the deposition temperature increased the grain size prior to the grain coalescence, reduced the number of gaps to be closed, and impeded the tensile stress generation during grain coalescence.

Secondly, raising the deposition temperature can promote compressive stress generation during the continued growth of the film. Compressive stress generation is attributed to the incorporation of excess atoms deposited on the surface into grain boundaries [13]. Raising the deposition temperature increased the diffusion coefficient and enhanced the diffusion and inserting of adatoms into the grain boundaries, hence promoting compressive stress generation.

During the subsequent long-term complex process, the residual stress increased during the TH (80 °C) and fell during NP for all the Au films deposited at different temperatures. In the 80 °C TH process, the growth of tensile residual stress is attributed to the volume contraction caused by closure of grain boundary voids and reduction of other defects in the as-deposited Au films [8]. According to the SEM micrographs, by increasing the deposition temperature, the original defects of the as-deposited films were largely reduced, thereby weakening the defect-reduction process, and resulting in less volume shrinkage and a smaller increase in tensile stress.

In the NP process, the stress relaxation (decrease) was considered to be caused by atom diffusion along the surface and grain boundary [8]. Lowering the residual stress reduced the driving force of the diffusion process. Meanwhile, increasing the grain size lengthened the diffusion distance, further weakening the diffusion process. Thus, the stress relaxation was reduced during the NP process. 

### 4.3. Comparisons between Post-Deposition Annealing and Increasing Deposition Temperature

**Figure 6:**
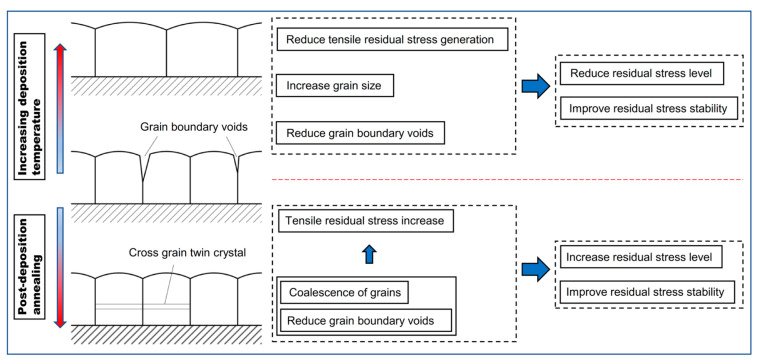
Comparison between increasing deposition temperature and post-deposition annealing.

Both post-deposition annealing [28] and increasing the deposition temperature can improve the residual stress stability. The comparisons between increasing deposition temperature and post-deposition annealing are schematically expressed in Figure 6. Based on microstructure analysis, the reason for enhancing the residual stress stability is quite similar. After post-deposition annealing, the grain boundary voids were greatly reduced and grain coalescence occurred through the formation of cross-grain twins [28]. The post-deposition annealing reduced the instability factor in the Au film and contributed to a more stable microstructure. This is the reason for the improvement of residual stress stability in the TH process. Additionally, the grain coalescence and formation of cross-grain twins impeded the grain boundary diffusion, which lead to the improvement of residual stress stability in the NP process. However, during the post-deposition annealing process, the film was confined by the substrate, reducing the grain boundary voids and other defects, leading to volumetric contraction and tensile stress level increasing simultaneously. There is causality between the microstructure stabilization process and the increase in tensile residual stress.

Increasing the deposition temperature can also reduce the grain boundary voids and other possible defects, resulting in a more stable microstructure and improving the residual stress stability. Though there is some association between the stress and the microstructure, the microstructure difference is not the direct cause of the stress difference. Due to a series of processes discussed in Section 4.1 and Section 4.2, reduction of the structural instability factor and decrease of the residual stress level can be achieved simultaneously during the deposition process.

## 5. Conclusions

In this paper, we studied the effects of deposition temperature on the long-term residual stress evolution of Au film. Au films were deposited under three different temperature conditions. The NP and TH combined process, simulating the long-term and complex conditions of actual operation, was performed on the Au films. The residual stress variation of Au film was studied and the mechanisms were discussed based on microstructure analysis. The following conclusions can be drawn:

(1) The Au films become more compact with the increase in deposition temperature. The grain size increases and the number of grain boundary voids decreases.

(2) Increasing the deposition temperature can decrease the initial tensile residual stress in the Au film.

(3) By increasing deposition temperature, the residual stress stability can be improved while reducing the residual stress level. Appropriate increase in the deposition temperature is an effective means to regulate the long-term stress stability of Au films.

## Figures and Tables

**Table 1:** Arrangement of the complex process consisting of TH and NP.

	NP1	TH1					NP2
Condition	NP	TH	TH	TH	TH	TH	NP
Time (day)	30	1	1	1	1	1	40

TH: simulating the service condition, 80 °C. NP: simulating the off-work state, room temperature.

**Table 2:** Residual stress variation value for Au films deposited under different temperature.

Process	Residual Stress Variation (MPa, Absolute Value)		
	No Preheating	130 °C Preheating	180 °C Preheating
NP1	46 ± 4	33 ± 2	28 ± 5
TH1	46 ± 8	34 ± 2	24 ± 2
NP2	19 ± 4	13 ± 5	8 ± 1
The entire process	48 ± 5	35 ± 0.5	29 ± 5

**Table 3:** The parameters used in the derivation of thermal stress.

Parameter	Value
$T_{r}$, room temperature	25 °C
$T_{d}$ for case of no preheating	98 °C
$T_{d}$ for case of 130 °C preheating	149 °C
$T_{d}$ for case of 130 °C preheating	180 °C
$\alpha_{f}$, CTE for Au [33]	14.16 × 10^−6^ °C^−1^
$\alpha_{s}$, CTE for silica [33]	0.55 × 10^−6^ °C^−1^
$E_{f}$, Young’s modulus of Au [33]	78.5 GPa
$\vartheta_{f}$, Poisson’s ratio of Au [33]	0.42

## Data Availability

The data presented in this study are available on request from the corresponding author.
